# Protocol for a systematic review of time to antibiotics (TTA) in patients with fever and neutropenia during chemotherapy for cancer (FN) and interventions aiming to reduce TTA

**DOI:** 10.1186/s13643-019-1006-8

**Published:** 2019-04-03

**Authors:** Christa Koenig, Jess Morgan, Roland A. Ammann, Lillian Sung, Bob Phillips

**Affiliations:** 1 Division of Pediatric Hematology/Oncology, Department of Pediatrics, Inselspital, Bern University Hospital, University of Bern, Bern, Switzerland; 2 Centre for Reviews and Dissemination, University of York, York, UK; 3 The Hospital for Sick Children, University of Toronto, Toronto, Ontario Canada; 4 Leeds Children’s Hospital, Leeds, UK

**Keywords:** Fever, Neutropenia, Cancer, Time to antibiotics

## Abstract

**Background:**

Fever and neutropenia (FN) is a common complication of chemotherapy for cancer. Prompt empiric broad-spectrum antibiotic therapy in FN is typically considered standard of care, but the definition of prompt is not clear. We seek to systematically review the available data on the association between time to antibiotics (TTA) administration and clinical outcomes in patients with FN being treated with chemotherapy. There have been several efforts to reduce TTA in patients with FN, by implementing specific interventions, presuming there will be a beneficial effect on patient-important outcomes. This systematic review will also collect data on such interventions and their effect to reduce TTA and potentially change clinical outcomes.

**Methods/design:**

The search will cover MEDLINE, MEDLINE In-Process & Other Non-Indexed Citations, EMBASE, CINAHL, CDSR, CENTRAL, and LILACS. A full-search strategy is provided. Lists of studies identified by references cited and forward citation searching of included articles will also be reviewed. Studies will be screened, and data extracted by one researcher and independently checked by a second. Confounding biases and quality of studies will be assessed with the risk of bias in non-randomised studies-of interventions (ROBINS-I) tool.

Data will be presented in narrative and tabular forms; in addition, if appropriate data is available, random effects meta-analysis will be used to examine TTA.

A detailed analysis plan, including an assessment of heterogeneity and publication bias, is provided.

**Discussion:**

This study aims to evaluate the association between TTA and patient-important clinical outcomes. Additionally, it will identify, critically appraise, and synthesise information on performed interventions and its effect to reduce TTA as a way of gaining insight into the potential use of these approaches. This will provide better knowledge for an adjusted treatment approach of FN.

**Systematic review registration:**

PROSPERO [CRD42018092948]

**Electronic supplementary material:**

The online version of this article (10.1186/s13643-019-1006-8) contains supplementary material, which is available to authorized users.

## Background

**Fig. 1:**
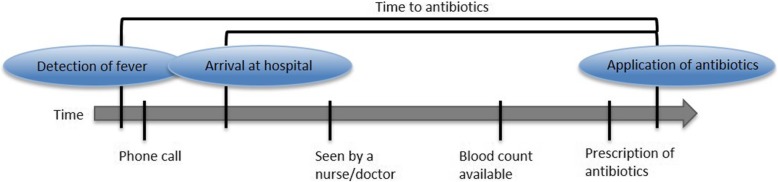
Scheme: time to antibiotics in patients with fever in neutropenia

Fever in chemotherapy-induced neutropenia (FN), if due to infection, is the most frequent potentially lethal complication of chemotherapy for cancer [1]. When absolute neutrophil count (ANC) drops below 0.5 × 10^9^/l, the risk of life-threatening bacterial infection increases [2]. Time to antibiotics (TTA) usually refers to the amount of time passed from arrival at the hospital to start of intravenous antibiotic administration [3–5]. Different definitions are sometimes used, for example, time from the first detection of fever [6] (Fig. 1).

Delays in presenting to medical care with fever may be one of the main reasons for poor outcome in FN; nevertheless, current European and American guidelines for treatment of FN in adult patients with cancer recommend administration of empiric broad-spectrum antibiotics within 1 h from the admission of a patient with FN [7, 8]. International FN guidelines for paediatric patients, developed by an international panel of experts, do not specify a target TTA [9]; the current German paediatric guidelines for treatment of FN recommend administration of antibiotics within 60 min without giving specific evidence [10]. Some organisations have defined TTA < 60 min as a measure of quality of care [5], but no systematic review has investigated the association of TTA and outcome in cancer patients.

Recommendations are based mainly on studies involving immunocompetent subjects with severe sepsis. In adult patients with severe sepsis [11] and meningitis [12, 13], delay in antibiotic administration is associated with a decrease in survival. After the onset of shock, there has been reported an increase in mortality of 7.6% for each hour delay [14]. Although strong and pathophysiologically sound, this association cannot directly be extrapolated to patients undergoing chemotherapy for cancer.

Damage to the gastrointestinal mucosa caused by anticancer agents provides a portal of entry for pathogenic bacteria, and the frequent need for indwelling central venous catheters allows for colonisation by an entry of Gram-positive skin flora [6]. These and other factors alongside chemotherapy-induced immunodeficiency predispose patients with cancer to bacteremia. Antibiotic treatment is initiated relatively early in all patients, unlike immunocompetent adults and, in contrast to the significantly ill patients that were examined in sepsis studies, fever is often the only clinical sign for infection. Overlapping parameters due to the impact of chemotherapy, e.g. therapy-induced thrombopenia, anaemia, or liver dysfunction, complicate detection and potentially outcomes of severe infections in cancer patients differently and may mean direct comparisons are inaccurate.

In summary and particularly in paediatric oncology there is a lack of evidence for the impact of TTA on outcome in FN.

A systematic review and meta-analysis will help to settle the controversies of conflicting studies, as well as to identify gaps in the current research and areas for further study.

Evidence identifying the importance of TTA is needed for an adjusted treatment approach and optimal TTA. If shown to be of low value, focus on other aspects of the treatment pathway than time, e.g. a rigorous diagnostic work, could improve quality of treatment.

### Aims, objectives, and overview of approach

This systematic review aims to evaluate the association between TTA and patient-important clinical outcomes and to explore the effect of important covariates on modifying outcomes in patients with FN during chemotherapy for cancer. We aim to define what TTA can be considered safe with regard to outcome. Additionally, the review will provide more detailed information about how important covariates of TTA are correlated with the outcome of FN episodes.

**Fig. 2:**
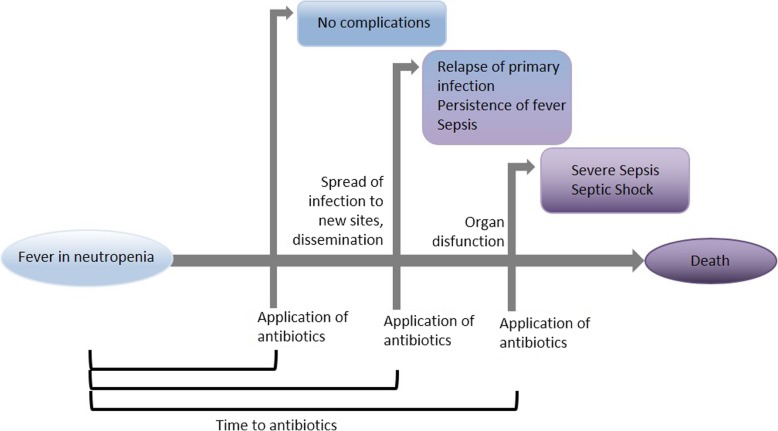
Scheme: pathophysiological model for different times to antibiotics

Drawing on pathophysiological models and previous work [15], we hypothesise shorter TTA will lead to reduced severe clinical illness, with less need for organ support, critical care, and reduced mortality rates. It has to be evaluated whether this is an accurate hypothesis for well-appearing patients or only for patients presenting already critically ill. The prompt treatment of infection may also lead to a reduced probability of dissemination of infection, and so shorter duration of fever, fewer relapses of infections or recurrences of fever, and shorter periods of hospitalisation for FN treatment (Fig. 2). We do not expect that TTA should be associated with the chance of identifying bacteraemia in initial investigations, as a bloodstream infection will either be present or not.

Several groups have attempted to reduce TTA in patients with FN during chemotherapy for cancer by implementing specific intervention in emergency departments (ED) and oncology wards. This systematic review will also identify, critically appraise, and synthesise information on interventions performed and their effect on TTA reduction in order to gain insight into the potential use of these approaches. If data exists, we will evaluate the impact of interventions that reduce TTA on important outcomes.

The close relationship between these two objectives justifies their combination. They are likely to be discovered during the same electronic search strategies and some studies will address both questions, saving duplication.

## Methods/design

This protocol specifies the conduct and reporting of a systematic review and meta-analysis in compliance with the guideline Preferred Reporting Items for Systematic Reviews and Meta-analyses (PRISMA) [16]. The systematic review will be undertaken following guidelines from the Cochrane Collaboration [17] and the Centre for Reviews and Dissemination (CRD) of the University of York [18]. The protocol for the review is registered in the PROSPERO Database [CRD42018092948]. The work for this review started in May 2018, and its publication is planned for summer 2019.

As this study is a systematic review of primary studies, no ethical approval is required.

### Search and retrieval strategy

Electronic sources will be searched for relevant studies including MEDLINE, MEDLINE In-Process & Other Non-Indexed Citations , EMBASE, CINAHL, CDSR, CENTRAL, and LILACS.

Lists of studies identified by references cited and forward citation searching (using ‘cited by’ in Google Scholar) of included articles will also be reviewed. The search strategy will include the Medical Subject Heading terms and text words to identify fever and neutropenia and the intervention of antibiotics. Antibiotics will also be searched by groups and names of antibiotic drugs (e.g. penicillins, beta-lactams, quinolones).

As a decision of balance between sensitivity and feasibility, EMBASE searches ‘time’ will be added as a required search factor to narrow the results. As most of the studies in neutropenic patients are cancer patients, we will not include a cancer filter in our search strategy. The full-search strategies are included in Additional file 1: Appendix 1.

The study selection process will be piloted by applying the search strategy to a sample of five papers in order to check that the correct papers would be identified.

Published and unpublished studies will be sought, and no language restrictions applied. The latter is important because we suspect that there may be a number of studies that have been performed in Spain, Portugal, and South America as these areas have active research in paediatric oncology FN research. Non-English language studies will be translated if this is possible within 3 months of running the searches; if unable to translate, this will be noted. This time limit will ensure that the results of this review are available to inform further aspects of an overarching PhD project. Where two publications reporting the same study exist, the one with the outcomes matching the review outcomes will be selected.

Authors of relevant studies and prominent clinicians within the field will be contacted as time allows seeking further studies, as this is likely to be a poorly indexed area of biomedical research (see Additional file 1, Appendix 2). If authors or the contacted clinicians provide unpublished or additive data beneficial for answering our review question, this data will be included.

The Society of Infectious Diseases of America published the first practice guidelines for the use of antimicrobial agents in neutropenic patients with fever in 1997 [19]. Since then, the standard management for all patients developing a fever while neutropenic has been empiric antibiotic treatment. Before this, practice was less consistent; therefore, studies from 1997 onwards will be included in this review.

### Screening for eligibility

The decision on the inclusion of a study will be made initially by screening the titles and abstracts of retrieved papers against the inclusion criteria by one reviewer (CK) to identify potentially relevant papers. After reading the full text of all potentially eligible studies, the final decision on whether to include them in the review or not will be made. A second reviewer will independently screen a sample of 50% of the retrieved papers. The kappa statistic for agreement will be calculated, and if this shows significant disagreement (κ < 0.4), all titles and abstracts will be screened by the second reviewer. In case of disagreement for inclusion, a consensus decision will be reached after discussion and if necessary by recourse to an independent adjudicator.

### Inclusion and exclusion criteria

Studies will be included if they meet the following criteria:

#### Study designs

It is not possible to do TTA randomisation, and it is not anticipated that many controlled studies will be available for both of our review questions. Therefore, observational studies, such as cohort studies and case-control studies are eligible for inclusion as well. In practice, the partial implementation of a time-reducing method may not be possible in one institution. So quasi-experimental studies as before-and-after studies and interrupted time series studies and studies with quality improvement methodology will be included. Studies examining the effect of attempting to reduce TTA are only included if they provide data from a comparison group.

Studies included may be retro- or prospective. Case reports are excluded from the review, owing to the high potential for bias in this study design.

Published studies, conference abstracts, and interim results will be included, but excluded if they provide insufficient data.

#### Population

Human participants who are receiving treatment for cancer (including leukaemias), presenting with FN will be included.

Combining results from adult and paediatric patients will enhance the number of patients and improve the validity of the analysis. Even when adult data may not be directly transferable to paediatric patients due to higher incidence of comorbidities, a different spectrum of cancer diseases and often less intense chemotherapy, drawing the reports together and synthesising their results will add to our understanding of TTA. It may allow broader analysis of confounding factors and increase the chance of discovering important covariates.

#### Interventions and comparators

Treating FN with any antibiotics will be the only required treatment criteria. For studies examining the association of TTA with outcomes, no additional intervention or comparator will be required as long vs. short TTA will be the comparator. If patients are included who have not been given antibiotics for the index episode, these will be noted but their data cannot be included in an analysis of TTA. It is likely that there is a difference within the association of TTA and clinical outcomes in TTA starting at detection of fever, compared to TTA starting at arrival at the hospital. TTA will be collected regardless of the used definition but the differences will be considered in discussion.

We are aware there are a variety of treatment regimes including IV therapy, oral therapy, and outpatient therapy. This creates a challenge as TTA may be influenced by the way of administration of antibiotics, e.g. oral treatment can be started faster, given that there is no need for intravenous access. Exclusion of oral treatment could overestimate the effect of longer TTA on outcome, because oral treatment, and for this reason, potentially shorter TTA, is more often given to low-risk patients. In contrary, it could underestimate the effect of TTA if IV antibiotics are more effective and therefore result in less adverse outcomes. Since we want to have a look at TTA as an independent variable regarding the outcome, it is justified to include all methods of administration in one review.

There are numerous different antibiotic regimes, in both adult and paediatric protocols [7–10]. The coverage of these antibiotics is certainly less varied than the specific antibiotics used: thus, differences between regimes are more likely to be related to the route of administration, including absorption and dosing, than the specific antibiotic used.

There will be no eligibility restriction concerning the definitions of fever and neutropenia but as they could influence TTA and outcomes they will be extracted and evaluated when comparing and pooling results.

Studies investigating an intervention or combination of interventions with the aim to reduce TTA in patients with cancer and FN will be included. Interventions can be implemented in inpatient or outpatient settings, performed by any person included in the FN management (e.g. nurses/physicians/patients/parents), and patient information/education would also be an included intervention. Interventions may be grouped during analysis (e.g. process changes, educational change) if considered appropriate.

There must be a comparison group cared for in the same way in the setting and with the same treatment regimens, except for the intervention studied. The comparison group can be of the same cohort and may be observed simultaneously or successively.

### Outcomes

There is marked variability in FN outcomes that are collected and reported [20]. An international collaborative group developed core outcomes [21] for febrile neutropenia research; the outcomes that should be collected within studies in FN are death, serious medical complication (admission to intensive care unit (ICU), severe sepsis, including septic shock), and potentially other outcomes like bacteremia, clinically or microbiologically documented infection, all-cause 30-day mortality, relapse of primary infection. These comprise the key outcomes which are patient, clinician, and research important and an effort will be made to examine these as key composite outcomes.

As this core outcome set is relatively new, the published outcomes will likely not be reported in full, and so the inclusion of studies will not be restricted to those who report these in detail. We expect studies which are likely to be included to have generally reported composite outcomes, possibly using different elements, rather than individual medical complications. Former multinational guidelines have recommended that the primary outcome of studies into FN should be such a composite measure [22]. The analysis in the review will necessarily be based on the definitions of outcomes within the original studies; these definitions will be collected alongside the outcome data.

#### Primary outcomes

There will be three primary outcomes in this review: safety and treatment adequacy (for all studies) and time to antibiotics (for those studies evaluating interventions).

#### Safety

Exploration of safety will consider death and serious medical complication (admission to ICU, severe sepsis, including septic shock) as a primary outcome. Knowledge about the safety of TTA is essential to be able to consider any adjustment for the treatment approach for FN.

#### Treatment adequacy

Delay in antibiotic administration may lead to dissemination and protraction course of an infection. Clinical signs of a protracted infection may be a relapse of primary infection and persistence of fever for more than 5 days after the start of treatment or recurrence of fever without a new infection. We will use treatment adequacy as a composite outcome to see whether a shorter TTA produces better treatment efficiency. Certainly, treatment adequacy is influenced by other variables as well, which will be explored in the secondary outcomes.

To be included, a study has to have recorded and provided data for one or more of the elements of the primary outcomes. We acknowledge that each study is unlikely to select an outcome that completely fits the definition given above. Therefore, the composite outcome that each individual study selects will be recorded within the data collection stage of this systematic review, and it may be only specific outcomes (for example, death, or admission to ICU) are reported in the different studies.

For studies which examine an intervention to assess its effects, there will be a further primary outcome assessed:

#### Time to antibiotics

For the analysis of the effect of specific interventions, the absolute reduction of TTA will be the primary outcome (process measure).

#### Secondary outcomes

As secondary outcomes, the individual components of safety and treatment adequacy will be analysed separately. For the analysis of the effect of specific interventions, the described primary and secondary outcomes of FN will be analysed as secondary outcomes.

Secondary outcomes will also include outcomes which may allow us to understand confounding. These are microbiologically defined infection, new infections, and modification of antibiotics and measurements of TTA other than absolute. We expect that these outcomes are not affected due to faster administration of antibiotics, but they may be influenced by the same covariates that influence treatment adequacy. As such, they will be used as negative controls for the association of TTA on safety and adequate treatment.

Additionally, days of fever and days of hospitalisation will be assessed. These may be influenced by the adequacy of initial antibiotic treatment and will not be regarded as negative controls for the association of TTA on safety and adequate treatment.

### Data extraction and assessment of risk of bias

Data will be extracted by one researcher (CK) using a standardised data extraction form (see Additional file 1, Appendix 3) and independently checked by a second (RAA). Intervention characteristics will be collected according to the Cochrane Effective Practice and Organisation of Care Review Group (EPOC) data collection checklist [23]. If the data to be extracted is unclear or not specific enough, authors will be contacted for further information. If there is no response, a further attempt to make contact will be made a fortnight later. If there is no response after a further 4 weeks, the data will be presumed unavailable. No data imputation is planned for missing data, and thus, studies with missing data will not be included in specific syntheses.

We expect several confounding factors. These may be patient-related factors as age, comorbidities, and initial illness severity. More rapid treatment of patients at higher risk will generate a triage bias. In contrary, the way of administration of antibiotics can create an inverse triage bias, as oral treatment for low-risk patients can be started faster. Structural factors as setting at FN diagnosis, localisation, and time of presentation may be important confounders as well, influencing TTA and outcome. The risk of bias in non-randomised studies–of interventions (ROBINS-I) tool [24] will help to assess biases, quality of the studies, and plan analysis appropriately.

Furthermore, a publication bias is expected, as successful interventions are more likely to be published than unsuccessful ones. Where possible, funnel plots of the study outcomes will be used to explore this. Expecting substantial in between-study heterogeneity, Rücker’s method [25] will be used as asymmetry test, if more than 10 studies are included in the meta-analysis [26].

### Methods of analysing/synthesis

Key study characteristics, study quality and the interventions aiming to reduce TTA will be described and summarised in narrative and tabular forms.

If it is considered appropriate (based on clinical and statistical homogeneity and the necessary data being available), meta-analysis will be undertaken in order to examine whether TTA is associated with safety, treatment adequacy, and how these are influenced by potential confounders. It is likely that any pooled analysis will only be possible with a subset of studies.

TTA will be categorised as within 1 h or more than 1 h, because current guidelines recommend administration within 1 h after arrival at the hospital [7, 8, 10]. Other definitions and analysis will be used, such as hourly increments or continuous TTA, within a sensitivity analysis. Results of studies with TTA starting at detection of fever will not be pooled with studies with TTA starting at arrival at the hospital. In the narrative analysis, TTA definition will be described and examined as a possible source of variation.

Odds ratios and 95% confidence intervals (CI) will be calculated or extracted for binary outcomes, standard mean difference (SMD), and 95% CI for continuous outcomes. The chosen effect measures will then be combined using a random effects model, given the anticipated clinical heterogeneity and reported with 95% confidence interval of the estimate and 95% prediction interval of the potential results found in future studies. All calculations will be done using the ‘R’ statistical environment. Forest plots will be presented for each outcome. For studies that provide them, data which are adjusted for covariates (e.g. clinical features suggestive of poor clinical outcome), will be pooled separately from unadjusted estimates.

Narrative synthesis will be undertaken to examine the association of TTA and outcome considering the risk of bias and potential mechanisms which lead to heterogeneous outcomes where data pooling is not appropriate.

#### Heterogeneity

Heterogeneity will be explored through consideration of study populations, study quality, and outcomes chosen. If possible, it will be quantified by using *I*^2^ (< 40% representing unimportant, 30 to 60% moderate, 50 to 90% substantial, and 75 to 100% considerable heterogeneity [17]), assessed with the Cochran’s *Q* test (chi-squared test, *p* value < 0.05 considered significant) and visualised by forest plots [17].

## Subgroups

### Subgroup analyses

A priori specified stratified analysis (if sufficient studies) is planned by:

#### Adult vs. paediatric

Due to differences in the disease spectrum, applied chemotherapy, incidence of comorbidities, and constitutional differences, results in adults cannot be directly transferred to children. Subgroup analysis will allow reasoning about differences in adults and children and whether it makes sense to use guidelines for adults in paediatric patients and vice versa. It will help to define reasonable recommendations for each group of patients. Age less than 18 years at FN diagnosis will be the definition for a paediatric patient or as defined by authors of the original studies.

#### High risk vs. low risk

Patients with FN are a heterogeneous population, with only a small proportion developing a serious medical complication. Studies who distinguished patients through initial risk stratification will allow separate analysis according to different risk groups. This will help to identify and minimise bias due to administration route, owing to the simpler and therefore more rapid treatment with oral antibiotics in low-risk patients and triage bias because severely ill patients are more likely to receive rapid treatment than patients in a good general condition. We will use the study’s definition to define the risk groups.

#### Severe neutropenia vs. non-severe neutropenia

An absolute neutrophil count (ANC) of < 0.5 × 10^9^/l is considered to be severe neutropenia [8] and most of the time the level used as definition for FN. The ANC level has been identified as factor for risk stratification in FN [9] and is used to define duration of therapy [7]. In this review, different definitions for neutropenia will be captured and some studies may include patients with non-severe neutropenia (ANC > 0.5 × 10^9^/l). Sub-group analysis will enable to distinguish between patients with non-severe neutropenia where antibiotics are not needed and therefore TTA does not make a difference and those with severe neutropenia, where more rapid treatment may have an influence on clinical outcome.

#### Comorbidities vs. no comorbidities in adult patients

Adult patients are more likely to have comorbidities. A separate analysis of patients without comorbidities is a more appropriate approach to compare adult data with paediatric patients. Relevant comorbidities must be present before admission for FN and may be any presence of a major abnormality in regard to organ dysfunction (for example, renal failure), comorbid conditions (for example, previous stroke), as demonstrated potentially through abnormal vital signs, clinical signs or symptoms, and laboratory or imaging data.

They will be collected and patients grouped into no comorbidities and with comorbidities defined as having any or multiple of the listed comorbidities.

#### Antibiotic prophylaxis vs. no prophylaxis

Administration of antibiotic agents to patients after chemotherapy, be it with or without neutropenia, without any suggestive signs or symptoms, reduces the incidence of infection and in some studies the infection-related mortality [27]. Positive microbiological detection rates by standard blood cultures vary depending on whether or not patients have received prophylactic antibiotics [7].

Centres that do not use the commonly given Fluoroquinolone prophylaxis report a predominance of Gram-negative bacteria [7] and usually Gram-positive infection result in a lower mortality than Gram-negative bacteria. A subgroup analysis will allow a more accurate conclusion on the effect of TTA on outcome and whether its impact is the same for patients who receive prophylaxis versus patients who do not.

#### Inpatients vs. outpatient FN

It is likely that patients with inpatient episodes of FN receive antibiotics faster than outpatients due to accessible IV line, no need for transport to the ED and no waiting time due to capacity constraints in EDs. Conversely, the reason for being an in-patient, such as the intensity of chemotherapy and general clinical condition may be important covariates for outcome. As these elements may differ significantly between out- and inpatients a subgroup analysis is sensible.

#### Localisation of presentation (emergency department vs. oncology unit)

One study has shown presentation to the ED having a longer TTA than when patients are admitted directly to the oncology unit, and presentation to the ED increases the risk of poor outcome [4]. This may be attributed due to high patient volumes in the ED setting, experience of personnel or different hours, and reasons for presentation (e.g. delayed detection of fever at night). Examining this across multiple studies will test this hypothesis further.

#### Admission time (night vs. day and weekend vs. rest of the week)

Several studies had shown that out-of-hours admission to hospital may increase patient mortality or morbidity [28, 29]. There are differences in emergency processes when we compare out-of-hour to working hours. Waiting-time and conduction of laboratory results may be longer on weekends and evenings and consultation with an oncologist may take more time.

Discrete analysis of admission times will show whether there are differences in TTA and can help to identify sources of delays and reasons for unsuccessful intervention approaches. This will be interesting and helpful when examining which interventions are effective and sensible for reduction of TTA.

#### Low/middle-income country vs. high-income country

We expect different barriers to providing rapid access to antibiotics and potentially different covariates influencing TTA among low/middle- and high-income countries. A study in a lower middle-income country has identified longer travel times, illiteracy, and poverty to be associated with delays in treatment of fever in paediatric leukaemia [15]. These variables may matter less in high-income countries. Subgroup analysis will allow us to explore differences and will be useful for future the planning of effective TTA reducing interventions in different developed countries.

### Methods of dissemination

The results of this review will be written for publication in a scholarly journal following the PRISMA reporting guidelines as closely as possible [16]. Areas of uncertainty and suggestions for further research will be outlined within the final report.

## Discussion

To make recommendations for targeted TTA, it is important to know whether the chosen timespan is safe and whether earlier antibiotic treatment can reduce complications of infections. In an effort to examine these key outcomes, we defined primary outcomes taking into account the views of patients, clinicians, and researchers as exemplified by the previously published core outcome data set [21]. The inclusion of studies will not be restricted to studies that report these in detail, as not all will report enough data for the defined primary outcomes. The chosen secondary outcomes will allow us to undertake a useful analysis even if a lack of reported primary outcomes exists.

We expect studies that are likely to be included to have generally reported composite outcomes, possibly using different elements, rather than individual medical complications. The analysis in the review will necessarily be based on the definitions of outcomes within the original studies; these definitions will be collected alongside the outcome data. In order to undertake the most comprehensive and accurate analysis possible, we will ask authors for more specific and fragmented data of used composite outcomes.

Specific limitations in the currently available data about TTA are expected, for example, the analysis of TTA in different clinically defined risk groups, e.g. high risk/low risk of adverse outcome of FN [9, 30]. Further, covariates that influence TTA (e.g. triage bias, waiting time for laboratory results, organisation streamlining) may be reported variably, and a comprehensive assessment would be valuable for the planning of future studies.

We will collect outcomes we do not expect to be affected by faster administration of antibiotics. They will be used as negative controls for the association of TTA on safety and treatment adequacy. Additionally, we defined a list of preferable subgroup analysis to embrace the complexity of important covariates.

In practice, TTA is often longer than the 60 min suggested in guidelines [3, 31]. Assuming that a shorter TTA leads to clinical benefits, several groups have tried to identify and reduce sources of delay by implementing interventions in EDs or hospital wards [3, 31–33]. Such interventions may include education of patients, implementation of staff consensus guidelines, high triage level for all patients with FN, and rapid rooming in of all patients with FN, alone or in combination. A systematic review collecting studies evaluating such interventions will help to identify reasonable interventions amenable to translation into clinical care and avoid repetition of unsuccessful approaches. It will also outline whether there is the need for further development of such interventions.

Besides the exclusion of case reports, there will be no restriction on study design. Although outcomes may change over time for reasons unrelated to the implemented intervention, we will include before-and-after studies even in the absence of interrupted time series analysis, what should be the primary analytic approach [34]. Before-and-after studies are confounded by time and may be triggered by a series of prior, unreported events, leaning to regression to the mean. Their effect on TTA has to be appraised carefully, but they will let us identify the range of interventions proposed and may offer possible solutions for individual local problems. As one goal is to describe and collect intervention strategies to disseminate knowledge or facilitate practice change to reduce TTA, exclusion of those would miss important information.

Collection of intervention characteristics according to the EPOC data collection checklist will allow us to classify interventions and make analysis more structured.

In summary, the findings from the review will be used to explore the implications of different TTA and TTA-reducing interventions, with the aim of informing future research and practice.

This will provide better knowledge for an adjusted treatment approach of FN in patients during chemotherapy for cancer.

## Additional files

Additional file 1: **Appendix 1:** Search strategy. **Appendix 2:** Email to elicit unpublished studies. **Appendix 3:** Data extraction form. (DOCX 60 kb)

## Abbreviations

ANC: Absolute neutrophile count
ED: Emergency department
FN: Fever and neutropenia
ICU: Intensive care unit
IV: Intravenous
PRISMA: Preferred Reporting Items for Systematic Reviews and Meta-Analyses
ROBINS-I tool: Risk of bias in non-randomised studies–of interventions tool
TTA: Time to antibiotics
