# Supplementary material for: Protocol for a systematic review of time to antibiotics (TTA) in patients with fever and neutropenia during chemotherapy for cancer (FN) and interventions aiming to reduce TTA
Source: Syst Rev. 2019 Apr 3;8:82. doi: 10.1186/s13643-019-1006-8 (PMC6446276; doi:10.1186/s13643-019-1006-8)
Supplement: Supplementary file 1 — Appendix 1: Search strategy. Appendix 2: Email to elicit unpublished studies. Appendix 3: Data extraction form. (DOCX 60 kb) [file 13643_2019_1006_MOESM1_ESM.docx]

**Appendix 1: Search strategy**

Ovid MEDLINE(R) In-Process & Other Non-Indexed Citations and Ovid MEDLINE(R) <1946 to Present>

1 Neutropenia/ or agranulocytosis/ or leukopenia/

2. exp Fever/ or exp “Fever of Unknown Origin”/ or exp Body Temperature/

3. 1 and 2

4. (febril* adj5 (neutropen* or granulocytop* or agranulocyto* or leukocytop??ni*)).ti,ab.

5. ((fever or temperature or temp) adj5 (neutropen* or granulocytop* or agranulocyto* or leukocytop??ni*)).ti,ab.

6. 3 or 4 or 5

7. exp anti-bacterial agents/

8. (antibiotic* or anti-biotic* or antibacterial* or anti-bacterial* or bacteriocid* or bactericid* or anti-mycrobacterial* or antimicrob* or anti-microb* ).tw.

9. exp beta-lactamases/ or exp beta-lactams/

10. exp penicillins/ or penicillin*.tw.

11. tazobactam*.tw

12. ureidopenicillin*.tw.

13. exp ticarcillin/ or ticarcillin*.tw.

14. exp piperacillin/ or piperacillin*.tw.

15. exp quinolones/ or quinolone*.tw.

16. exp ciprofloxacin/ or ciprofloxacin*.tw.

17. exp ceftazidime/ or ceftazidime*.tw.

18. meropenem*.tw.

19. exp imipenem/ or imipenem*.tw.

20. exp aztreonam/ or astreonam*.tw.

21. exp aminoglycosides/

22. aminoglycoside*.tw.

23. exp amikacin/ or amikacin*.tw.

24. exp gentamicins/ or gentam?cin*.tw.

25. exp tobramycin/ or tobram?cin*.tw.

26. exp kanamycin/ or kanam?cin*.tw.

27. exp netilmicin/ or netilm?cin*.tw.

28. (beta-lactam* or beta?lactam*).tw.

29 6 and (or/7-28)

**Appendix 2: Email to elicit unpublished studies**

Dear ­­______,

We are performing a systematic review about time to antibiotic (TTA) in adults and children with fever and neutropenia undergoing chemotherapy for cancer. In particular, we aim to investigate the association of TTA on patient important outcomes and to describe the effect of specific interventions aiming to reduce TTA in those patients. We are contacting you, as an expert within this field, to enquire if you know about any ongoing or unpublished work within this area. We are looking for studies that meet the following criteria:

Patients: Patients (adults and children) with fever and neutropenia during chemotherapy for cancer. Intervention (any of):

- Measured time to antibiotics (mostly defined as arrival at the hospital to first dose of antibiotics administration)
- Studies examining interventions attempting to reduce TTA

Study design: no restriction.

Outcome (any of):

- Adverse outcomes e.g.: death, admission to intensive care unit, severe sepsis (including septic shock), persistence of fever, recurrence of fever
- Reduction of TTA

Should you know of any appropriate studies, we would greatly appreciate if you could share this information. If you have any questions about whether a study might be eligible for inclusion within this systematic review, please do not hesitate to contact us.

Many thanks for your time and consideration,

Yours sincerely,

**Appendix 3: Data extraction form**

# Data Extraction: For each included study. For the systematic review of time to antibiotics (TTA) in patients with fever and neutropenia during chemotherapy for cancer (FN) and interventions aiming to reduce TTA.

# **General Information**

| - First author: |  | - Checked by: |  |
| --- | --- | --- | --- |
| - Extracted by: |  | | |
| - Title |  | | |
| - Type of report (i.e. abstract or full manuscript): |  | | |
| - Year of publication/Journal: |  | | |
| - Country: |  | | |
| - Number of centres included: |  | | |
| - Dates of participant recruitment: |  | | |
| - Study type: |  | | |
| - Objective of the study: |  | | |
| - Comparator: |  | | |
| - Inclusion Criteria: |  | | |
| - Exclusion Criteria: |  | | |
| - Definition for fever: |  | | |
| - Definition for neutropenia: |  | | |
| - Definition for TTA: |  | | |
| - Antibiotics given: |  | | |
| - Ethical approval: | - done  not clear  not necessary | | |
| - Power calculation |  | | |

# Participants

|  | - overall | - TTA <60min | - TTA >60min | - Subgroup | - (specify) | - (specify) | - (specify) |
| --- | --- | --- | --- | --- | --- | --- | --- |
| - Number of included patients: |  |  |  |  |  |  |  |
| - Number of included episodes: |  |  |  |  |  |  |  |
| - Average age (range, SD): |  |  |  |  |  |  |  |
| - Gender (M:F): |  |  |  |  |  |  |  |
| - Type of cancer: |  |  |  |  |  |  |  |
| - TTA (specify value/range) |  |  |  |  |  |  |  |
| - Location at FN diagnosis (out:in): |  |  |  |  |  |  |  |
| - Location of presentation (ED:Unit): |  |  |  |  |  |  |  |
| - AB prophylaxis (No:Yes) |  |  |  |  |  |  |  |
| - Route of antibiotics (IV: oral) |  |  |  |  |  |  |  |
| - Antibiotics given |  |  |  |  |  |  |  |
| - High risk - Definition used: |  |  |  |  |  |  |  |
| - Low risk - Definition used: |  |  |  |  |  |  |  |
| - Time to the hospital |  |  |  |  |  |  |  |
| - Comments: |  | | | | | | |

# Outcomes

| - Outcomes reported | - Please tick outcomes reported: | - Safety - death - admission to ICU - severe sepsis/shock - Treatment adequacy - persistence of fever - recurrence of fever/infection - Adverse event/clinical course - microbiologically defined infection - new infection - modification of antibiotics - days of fever - days of hospitalization | - TTA - Others (e.g. composite outcomes not split) - … |
| --- | --- | --- | --- |
| - Definition as stated in report/paper: - (Same as collected for ROBINS page 2). | |  |  |

# Subgroups

| - Analysis of subgroups/ confounder defined in protocol : | - no subgroups reported - Age - Risk group - Comorbidities - AB Prophylaxis - Location at diagnosis - Location of presentation - Admission time - Other (specify) | - Comments: |
| --- | --- | --- |
| Fill out the outcome questions for each subgroup reported. |  |  |
| Comments to additional data on subgroups: |  | |

#

# Primary Outcomes

**1. Safety**

**Death (Number of/ Out of)** not reported

- Overall
- TTA <60min
- TTA >60min
- Subgroup (specify)

**TTA (media or mean/CI or SD)**

- Death
- Survivor

**ICU admission (Number of/ Out of)** not reported

- Overall
- TTA <60min
- TTA >60min
- Subgroup (specify)

**TTA (media or mean/CI or SD)**

- ICU Admission
- No Admission

**Severe Sepsis/Shock (Number of/ Out of)** not reported

| - Definition as stated in report/paper: |  |
| --- | --- |

- Overall
- TTA <60min
- TTA >60min
- Subgroup (specify)

**TTA (media or mean/CI or SD)**

- Sepsis/Shock
- No Sepsis/Shock

**2. Treatment adequacy**

**Fever Persistence (mean duration of fever)** not reported

| - Definition as stated in report/paper: |  |
| --- | --- |

- Overall
- TTA <60min
- TTA >60min
- Subgroup (specify)

**TTA (media or mean/CI or SD)**

- “short” fever
- “persistent” fever

**Recurrence of fever/primary infection (Number of/ Out of)** not reported

| - Definition as stated in report/paper: |  |
| --- | --- |

- Overall
- TTA <60min
- TTA >60min
- Subgroup (specify)

**TTA (media or mean/CI or SD)**

- No recurrence
- Recurrence of fever

# Secondary outcomes

**Days of Fever (media or mean/CI or SD)** not reported

- Overall
- TTA <60min
- TTA >60min
- Subgroup (specify)

**Days of hospitalization (media or mean/CI or SD)** not reported

- Overall
- TTA <60min
- TTA >60min
- Subgroup (specify)

**Microbiologically defined infection (Number of/ Out of)** not reported

| - Definition as stated in report/paper: |  |
| --- | --- |

- Overall
- TTA <60min
- TTA >60min
- Subgroup (specify)

**TTA (media or mean/CI or SD)**

- Microbiologically defined infection
- No defined infection

**New infection (Number of/ Out of)** not reported

| - Definition as stated in report/paper: |  |
| --- | --- |

- Overall
- TTA <60min
- TTA >60min
- Subgroup (specify)

**TTA (media or mean/CI or SD)**

- New infection
- No new infection

**Modification of antibiotics (Number of/ Out of)** not reported

| - Definition as stated in report/paper: |  |
| --- | --- |

- Overall
- TTA <60min
- TTA >60min
- Subgroup (specify)

**TTA (media or mean/CI or SD)**

- Modification
- No modification

**Other (specify) (Number of/ Out of)** not reported

| - Definition as stated in report/paper: |  |
| --- | --- |

- Specify:
- Overall
- TTA <60min
- TTA >60min
- Subgroup (specify)

**TTA (media or mean/CI or SD)**

- With other Outcome
- Without other outcome

# Outcome Intervention

**TTA (mean/SD)** not reported

- Overall
- Baseline Pre-intervention
- Post-intervention
- Sub-processes (specify)

**TTA reduction**

- Difference pre-port Intervention:

**TTA < 60min not reported**

- Overall
- Baseline Pre-intervention
- Post-intervention
- Sub-processes (specify)

**Change in % of TTA <60min**

- Difference pre-port Intervention:

**If several groups/measured points:**

- Number of points pre and post
- Number of patients or measurement units (eg laboratory tests) in wholeseries
- Time interval between points
- Report absolute change in natural units
- Report percentage relative change
- Report the model used and statistical significance
- ***Is information on the value of individual observations over time only reported graphically in the original paper?*** yes  no

| Any effect in reducing TTA (as reported) | yes  no | |
| --- | --- | --- |
| If yes: | reduction absolute (min)  more % <60min | |
| Length of time during which TTA was measured after initiation of the intervention |  | |
| Length of post-intervention follow-up period |  | |
| Identification of a possible ceiling effect  e.g. there was little room for improvement in provider performance,  because it was adequate without the intervention | Identified by investigator  yes  no  not clear | Identified by reviewer  yes  no  not clear |
| Identified sources of delays TTA by the authors: (Before or after intervention) |  | |

##

# Interventions

According to The Cochrane Effective Practice and Organisation of Care Review Group (EPOC) data collection checklist (Use for clarification, there are not all point included, some are collected elsewhere, some unlikely to be important as reimbursement system and Quality assessment will be done with ROBINS-I.)

| Type of intervention: | | No intervention  Skip the rest of the form | Professional  Financial  Organisational  Provider orientated  Patient orientated  Structural  Regulatory |
| --- | --- | --- | --- |
| Description of intervention as stated in report/paper: | | | |
| Controls | no intervention control  standard practice (if different to no intervention)  another intervention | | |
| Setting of intervention | (specify)  in practice setting  not in practice setting  not clear | | |

| Type of targeted behaviour | Clinical prevention services  Diagnosis  Test ordering  Referrals (Überweisung)  Procedures  Prescribing  General management of a problem (e.g. the treatment of hypertension)  Patient education/advice  Professional-patient communication  Record keeping  Financial (resource use)  Discharge planning  Patient outcome  Other (specify)  NOT CLEAR |
| --- | --- |
| Provider  (see examples in Checklist page 15) | Profession:  Level of training:  Clinical speciality:  Age:  Time since graduation:  Number of included providers: |
| Location of care | inpatient  outpatient (e.g. ambulatory care)  mixed  not clear |
| Academic status of setting | University based/teaching setting (i.e. not simply university affilation)  Non-teaching setting  Mixed  Not clear |
| Proportion of eligible providers (or allocation units). Out of the total number in the sampling frame. |  |
| Unit of allocation (i.e. who or what was allocated to study groups)  Unit of analysis (i.e. results analysed as events per practice) | patients  provider  practice  Institution  community  firm  clinic day  other (spedify)  not clear |
| Investigators identified specific barriers to change in the target population | information management  clinical uncertainty  sense of competence  Perceptions of liability  Patient expectations  Standards of practice  financial disincentives  Administrative constraints  other  not done  not clear |
| **Characteristics of intervention** | |
| Evidence base of recommendation | done  not clear  not done |
| Purpose of recommendation | Appropriate management  cost containment  other  Not clear |
| Nature of desired change | new management  stopping new management  reduction of established management  increase established management  cessation of established management  modification of established management (e.g. increased management in one activity, reduction in another)  not clear |
| Format  (state the medium employed) | interpersonal  paper  audio/visual  computer /interactive  multiple media used  other  not clear |
| Source | local clinicians  local expert body  national professional expert body  national government expert body  international professional expert body  international professional government expert body  other (specify)  not clear |
| Intervention based upon implementation of clinical practice guidelines | done  not clear  not done |
| Clinical practice guidelines developed through formal consensus  process | done  not clear  not done |
| Recipient  State whether each intervention was delivered to: | individual  group  not clear |
| Deliverer  State who (or what) delivered the intervention (score all relevant) | Pharmacist  Local expert (state profession)  research worker  management representative  computer system  other (specify)  not clear |
| Timing | Proximity to clinical decision-making:  Frequency/number of intervention events: Duration of intervention: |
